# Molecular Dynamics Analysis of a Novel β3 Pro189Ser Mutation in a Patient with Glanzmann Thrombasthenia Differentially Affecting αIIbβ3 and αvβ3 Expression

**DOI:** 10.1371/journal.pone.0078683

**Published:** 2013-11-13

**Authors:** Michel Laguerre, Essa Sabi, Martina Daly, Jacqueline Stockley, Paquita Nurden, Xavier Pillois, Alan T. Nurden

**Affiliations:** 1 Institut Européen de Chimie et Biologie, Pessac, France; 2 Department of Cardiovascular Science, University of Sheffield, Sheffield, England, United Kingdom; 3 Plateforme Technologique et d′Innovation Biomédicale, Hôpital Xavier Arnozan, Pessac, France; 4 Centre Hospitalier Universitaire de Marseille, Hôpital Timone, Marseille, France; 5 Unité 1034 Institut National de la Santé et de la Recherche Médicale, Hôpital du Haut-Lévèque, Pessac, France; University of Leuven, Belgium

## Abstract

Mutations in *ITGA2B* and *ITGB3* cause Glanzmann thrombasthenia, an inherited bleeding disorder in which platelets fail to aggregate when stimulated. Whereas an absence of expression or qualitative defects of αIIbβ3 mainly affect platelets and megakaryocytes, αvβ3 has a widespread tissue distribution. Little is known of how amino acid substitutions of β3 comparatively affect the expression and structure of both integrins. We now report computer modelling including molecular dynamics simulations of extracellular head domains of αIIbβ3 and αvβ3 to determine the role of a novel β3 Pro189Ser (P163S in the mature protein) substitution that abrogates αIIbβ3 expression in platelets while allowing synthesis of αvβ3. Transfection of wild-type and mutated integrins in CHO cells confirmed that only αvβ3 surface expression was maintained. Modeling initially confirmed that replacement of αIIb by αv in the dimer results in a significant decrease in surface contacts at the subunit interface. For αIIbβ3, the presence of β3S163 specifically displaces an α-helix starting at position 259 and interacting with β3R261 while there is a moderate 11% increase in intra-subunit H-bonds and a very weak decrease in the global H-bond network. In contrast, for αvβ3, S163 has different effects with β3R261 coming deeper into the propeller with a 43% increase in intra-subunit H-bonds but with little effect on the global H-bond network. Compared to the WT integrins, the P163S mutation induces a small increase in the inter-subunit fluctuations for αIIbβ3 but a more rigid structure for αvβ3. Overall, this mutation stabilizes αvβ3 despite preventing αIIbβ3 expression.

## Introduction

Glanzmann thrombasthenia (GT) is a rare inherited disease of platelet aggregation caused by quantitative and/or qualitative deficiencies of the αIIbβ3 integrin [1]–[3]. The result is lifelong bleeding due to the inability of platelets to plug injured blood vessels. The *ITGA2B* and *ITGB3* genes that encode αIIbβ3 co-localize at chromosome 17q21.32 although their transcription is not coordinated [4]. Biosynthesis of αIIbβ3 occurs in megakaryocytes (MKs) in the bone marrow; anucleate platelets are released in large numbers from protrusions called proplatelets extruded into the blood circulation [5]. GT is given by a large variety of nonsense and missense mutations, gene rearrangements including small insertions or deletions, splice site defects and frameshifts that occur across the 45 exons that compose *ITGA2B* and *ITGB3* [2], [3]. Whereas αIIb is mostly confined to the MK lineage, β3 is also present as αvβ3, a major integrin of vascular, blood and tissue cells; in contrast, αvβ3 is a very minor component in platelets [6]–[8]. Mutations in *ITGA2B* are specific for αIIbβ3, but those effecting *ITGB3* extend to both β3-containing integrins and potentially concern all cell types expressing αvβ3. While a majority of *ITGB3* mutations affect β3 expression, missense mutations can have different effects on the capacity of β3 to interact with αIIb and αv. Indeed, rare β3 mutations have been shown to allow αvβ3 expression while preventing the formation and/or maturation of αIIbβ3. Alternatively, while permitting the expression of both integrins they may affect their function differently [9]–[13].

Elucidation of the crystal structures of the αvβ3 and αIIbβ3 extracellular domains has allowed a close investigation of the interactions at the head domain interface between β3 and αv or αIIb and has revealed distinct structural differences [14]–[19]. We now report studies that include a molecular dynamics analysis to investigate the effects on integrin structure of a novel β3Pro189Ser (P163S in the mature protein) mutation that we have located in a case of type I GT. This mutation prevents expression of the αIIbβ3 complex while stabilizing the interaction between β3 and αv.

## Materials and Methods

### Ethics Statement

Written informed consent was obtained from the patient prior to providing blood for the mutation analysis that was performed as part of the diagnosis of her disease. The patient herself reviewed her case report in the days preceding submittal of the manuscript. The study protocol was approved by the Human Research Ethics Committee of Alsace under the promotion of the French National Institute of Health and Medical Research (INSERM, Paris) under protocol RBM 04-14 for the French National Network for Disorders of Platelet Production and Function (Directors: JP Cazenave and AT Nurden) and was performed according to the Declaration of Helsinki.

### Subjects

**Figure 1:**
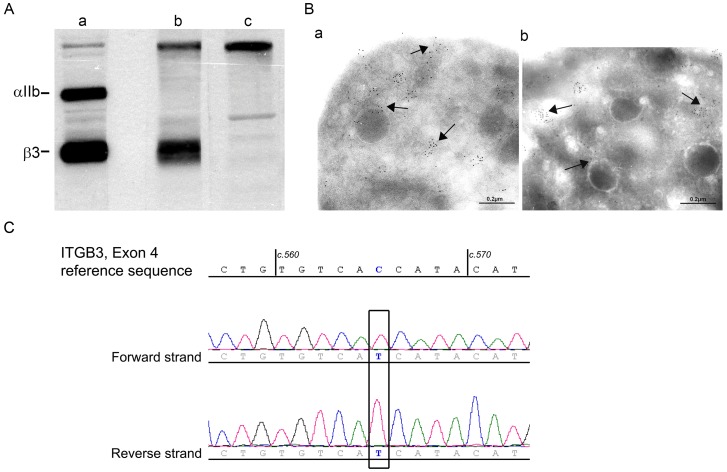
Initial studies characterizing the molecular defect of αIIbβ3 of the patient’s platelets. A/Western blotting of αIIb and β3 in samples of SDS-soluble extracts of (a) control platelets (5μg protein), (b) the patient under study (60 μg) and (c) a second type I GT patient [21] with a large *ITGB3* deletion preventing synthesis of β3 (60 μg). The integrin subunits were detected with a polyclonal antibody to αIIbβ3 with bound IgG located using ^125^I-labeled protein A. B/Immunogold labeling was performed on frozen-thin platelet sections using a pool of murine monoclonal antibodies specific for the αv subunit [8] and bound antibody located for platelets from (a) a control donor and (b) from the patient using a species-specific second antibody to mouse IgG adsorbed on 5 nm gold particles and electron microscopy. Arrows highlight the largely vesicular distribution of αvβ3. C/Direct sequencing of genomic DNA of the patient (forward and reverse strands) for exon 4 of *ITGB3*. The nucleotide concerned by the mutation is framed. The patient is homozygous for a c.565 C/T transition leading to a p.Pro189Ser substitution (P163S in the primary β3 structure nomenclature).

The propositus is a 49 year-old French woman of consanguineous parents who was diagnosed with GT when 5 years old (Case History S1). In brief, her platelets failed to aggregate with all physiologic agonists and failed to retract a clot. They minimally bound monoclonal antibodies (MoAbs) to αIIbβ3 in flow cytometry despite a normal presence of other membrane glycoproteins (Figure S1). αIIb was absent in western blotting performed using a polyclonal antibody to αIIbβ3 with bound immunoglobulin located using ^125^I-labeled Protein A as described [20]; however, residual β3 was present in low amounts and was of normal migration (Figure 1A). As a further control for the specificity of antibody binding, we also studied in parallel platelets of a patient with a large *ITGB3* deletion preventing β3 synthesis [21]. The residual β3 seen for the propositus suggested that αvβ3 was maintained, a finding confirmed for platelets by immunogold-labelling and electron microscopy performed according to our standard procedures [8]. It should be noted that αvβ3 is organized essentially in intracellular vesicles as first described by us both in normal platelets and in another type I GT patient [8].

### DNA analysis

Genomic DNA was extracted from 200 μl buffy coat (leukocyte-rich zone at the interface between platelet-rich plasma and red blood cells) of a centrifuged EDTA-anticoagulated blood sample, with a QiaAmp®DNA minikit (Qiagen S.A., Courtaboeuf, France) according to the manufacturer’s protocol. Direct sequencing of all exons and splice sites of *ITGA2B* (30 exons) and *ITGB3* (15 exons) was performed by the French National Sequencing Center (Génoscope, Evry, France). Briefly, exons and flanking regions of *ITGA2B* and *ITGB3* were amplified by polymerase chain reaction (PCR) with a high fidelity Taq polymerase permitting large fragment amplification (TaKaRa LA Taq® DNA Polymerase, Millipore SA, Molsheim, France). PCR fragments were sequenced using the BigDye Terminator v3.1 Cycle reaction kit (Life Technologies, Saint Aubin, France) and a 3730 DNA Analyzer from Life Technologies. Further details of the Methods including the structure of all oligonucleotides are available on request. Pathogenicity of mutations was analyzed using Alamut Mutation Interpretation Software (Seine Biopolis, Rouen, France).

### Transfection Studies

The QuikChange Lightning Site-Directed Mutagenesis Kit (Qiagen, Manchester, UK) was used according to manufacturer’s instructions to introduce the c.565C>T transition predicting the p.P189S substitution in β3 (P163S in mature β3) into a wild-type (WT) β3 expression plasmid, pcDNA3.1-WTβ3, to derive the mutated plasmid, pcDNA3.1-P163Sβ3.

Chinese hamster ovary (CHO) cells were cultured in Roswell Park Memorial Institute (RPMI) 1640-GlutaMAX™ (Gibco-Life Technologies, Paisley, UK) medium supplemented with 10% fetal calf serum. Cells were transiently transfected with empty plasmid (pcDNA3.1) or the WT-β3 or P163Sβ3 expression plasmid either alone, or along with a WT-αIIb expression plasmid, pcDNA3.1-WTαIIb. For each well of a 6 well plate, a total of 2 µg of plasmid DNA was diluted in 500 µl of serum-free medium, before adding 5 µl of Lipofectamine LTX (Invitrogen-Life Technologies, Paisley, UK) and incubating the plate at room temperature for 25 minutes to allow formation of Lipofectamine-DNA complexes. CHO cells, grown to 80–90% confluence, were passaged and 1x10^5^ cells, in 1.5 ml of complete medium, were added to each well, before incubating the plate at 37°C in the presence of 5% CO_2_. Forty eight hours after transfection, cells were harvested and expression of cell surface αIIbβ3 assessed by flow cytometry on a FACSCalibur flow cytometer (BD Biosciences, Oxford, UK) using fluorescein isothiocyanate (FITC) conjugated anti-CD41 (MCA467F; AbD Serotec, Kidlington, UK) and phycoerythrin (PE) conjugated anti-CD61 (BD555754; BD Biosciences) monoclonal antibodies. Intracellular β3 expression was assessed similarly after fixing and rendering the cells permeable using the BD Cytofix/Cytoperm Kit (BD Biosciences). The ability of WT and β3P163S subunits to bind to αv, expressed endogenously by CHO cells, was assessed using FITC conjugated monoclonal anti-αvβ3 (LM609; Chemicon, Chandlers Ford, UK).

### Static Modeling of αIIbβ3 and αvβ3

Models were obtained using the PyMol Molecular Graphics System, version 1.3, Schrödinger, LLC (www.pymol.org) and 3fcs and 1u8c pdb files for crystal structures of αIIb and αv in complex with β3 in the bent conformation. Amino acids are visualized in the rotamer form showing side change orientations incorporated from the Dunbrack Backbone library with the maximum probability [2], [3].

### Molecular Dynamics Simulations

For αIIbβ3 we started from the X-ray structure with PDB code 3NIG (resolution 2.25 Å) and for αvβ3 with PDB code 3IJE (resolution 2.90 Å). As β3 is very large and would have necessitated long simulation times we reduced the size of the structure examined. The GT database (http://sinaicentral.mssm.edu/intranet/research/glanzmann) shows that while a large majority of missense mutations are located in the “head-groups” of the two subunits, few are found at the N-terminal end of β3. Moreover, the distal extracellular β3-domain linked to the transmembrane sequence is free and being close to the α-subunit is prone to stick onto its surface during simulations leading to an abnormal complex. It was therefore decided to truncate β3 at residues 110 and 354. As a result, the extracellular N-terminal domain composing amino acids (aa) 1 to 110 and the membrane proximal C-terminal part represented by residues 354 to 466 were removed. The truncated β3 from P111 to S353 was used for αIIbβ3 and αvβ3.

In order to check that the truncation of β3 had no detrimental influence on the behavior of the complexes, the two wild-type (WT) assemblies were submitted to a long (60 ns) molecular dynamics simulation. For this, the protein complex was centered in a rectangular water box with dimensions: 110×100×100 Å. Then the whole system was neutralized and 150 mM NaCl added. This resulted in a box with 29,100 to 29,200 water molecules and approximately 184 NaCl molecules (the number may vary in the presence of the mutation). Calculations were accomplished using GROMACS 4.5 and the GROMOS96 force field (G43a1) packages [22]. The model for water was SPC (simple point charge). Molecular dynamics runs were performed at constant temperature (300 K, time constant for coupling τp = 0.1 ps) and pressure (P = 1 bar, τp = 0.5 ps) with a Berendsen coupling algorithm [23]. The time step = 2 fs, particle meshed Ewald (PME) method [24] was used with a cubic grid (1 Å), Van der Waals (VDW) cut off = 10 Å, and frames were saved every 1000 steps.

In the same way, the β3 mutant P163S was created via the appropriate module in Discovery Studio version 3.1 and the two mutated complexes were submitted to identical molecular dynamics runs. One simulation was performed on truncated β3 alone, either WT or with the P163S mutation. Here we used a smaller water box with dimensions: 80×80×80 Å containing around 15,700 water molecules and 93 NaCl molecules. The molecular dynamics simulations were identical to the large box. In trajectory analyses root mean square deviations (RMSD) and root mean square fluctuations (RMSF) were calculated on Calpha positions as described in Jallu et al [25].

## Results

### Molecular Characterization and Mutation Analysis

As shown in Figure 1A and Figure S1, platelets of the patient have a severe deficit in αIIbβ3 that is characteristic of type I GT [1]. Small amounts of residual β3 were observed by Western blotting and αvβ3 was normally localized in her platelets by immunoelectron microscopy (Figure 1B). This presence would suggest a genetic defect of *ITGA2B*. But unexpectedly, this was not confirmed by direct sequencing of *ITGA2B* (30 exons) and *ITGB3* (15 exons) and their splice sites with results showing a homozygous C to T transition at position 565 of the cDNA (c.565C>T) within exon 4 of the *ITGB3* gene. This gave a p.Pro189Ser substitution (P163S in the mature protein) (Figure 1C). No other potential pathological mutations were located in either gene. Of interest, genotyping for the HPA1a/1b alloantigen system (L33P in the mature protein) carried by β3 showed homozygosity for the rare β3 HPA-1b alloantigen, a finding restricted to about 2% of Caucasians [26].

β3P163 is highly conserved within mammals and vertebrates (Figure S2) and within different human β-subunits suggesting that it is important for integrin biosynthesis and/or function. According to the Alamut software, the physical and chemical deviation between a proline and a serine is important (Grantham score: 74) and according to the SIFT (sorting inherent from tolerant) score this mutation is predicted to be deleterious (SIFT score: 0.0).

### Expression Studies in CHO Cells

**Figure 2:**
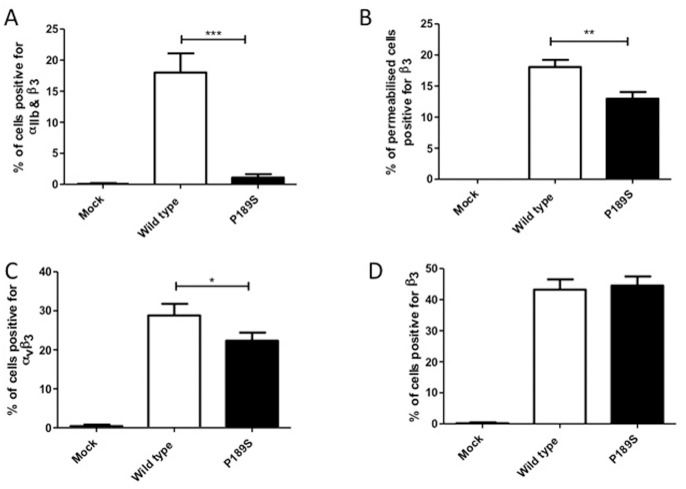
Expression of normal and mutated αIIbβ3 and αvβ3 in CHO cells. CHO cells were transfected with either wild type or mutated β3 P163S expression plasmids alone (C, D) or in the presence of wild type αIIb expression plasmid (A, B), or mock transfected with empty vector as a negative control. Forty-eight hours after transfection, the percentage of cells expressing both αIIb and β3 (A), αvβ3 (C) and β3 (D) were determined by flow cytometry. Intracellular expression of β3 was assessed after permeabilization of the cells (B). Data represent the mean and standard deviation of three independent experiments. ***p<0.001, **p<0.01, *p<0.05 as calculated by unpaired t-tests.

The potential pathogenicity of the P163S substitution in β3 was further investigated after introduction of the mutation into a β3 expression construct and transient expression in CHO cells, either alone to give rise to a chimeric complex with endogenous hamster αv, or with co-expression of normal human αIIb (Figure 2). There was a 94% reduction in surface expression of the αIIbβ3 receptor in CHO cells expressing P163Sβ3 compared to those expressing WTβ3 mirroring the deficit in expression of αIIbβ3 observed in platelets from the patient with the defect (Figure 2A). In contrast, assessment of β3 after permeabilisation of the cells revealed that the β3P163S subunit was expressed intracellularly at 71% of the levels of those of WTβ3 (Figure 2B) and staining of αvβ3 on CHO cells transfected with β3 alone indicated that P163Sβ3 was able to form a chimeric complex with hamster αv which was expressed at 78% of the levels of the chimeric complex of WTβ3 and hamster αv (Figure 2C). Moreover, labeling of cells transfected with the WT and P163Sβ3 subunits alone, using the monoclonal antibody to β3 to detect the chimeric complex of αvβ3, showed no difference between cells expressing WTβ3 and P163Sβ3 subunits (Figure 2D).

### Static Modeling Analysis

**Figure 3:**
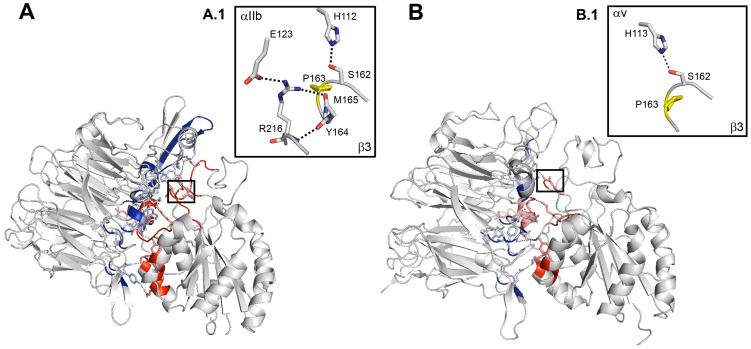
Static modeling showing the positioning of β3P163. Panel (A) represents computer-drawn ribbon diagrams of the WT αIIb and β3 headpiece complex and panel (B) the corresponding structure for WT αv and β3 subunits. Interacting surfaces are colored in blue for αIIb or αv, and in red for β3. Amino acids forming a H-bond with their counterpart in the other subunit are represented as sticks. H-bonds are shown as dotted lines. Interactions modified by the mutation are highlighted in boxes A.1 and B.1. The mutated proline is colored in yellow. Models were obtained using the PyMol Molecular Graphics System, version 1.3, Schrödinger, LLC and 3fcs and 1u8c pdb files for the crystal structure of αIIb in complex with β3 and αv in complex with β3 in bent conformations.

Crystallography showed that β3P163 residue is situated at the interface between the αIIb or αv and β3 subunit head domains [14], [17]. This is illustrated by static modeling showing the contacts (colored) between the wild-type (WT) headpieces of αIIb and αv (in blue) with β3 (in red) (Figure 3A and B). Strikingly, the adjoining β3S162 is also mutated in a case of GT underlining the importance of this sequence situated in the β-I domain (see Discussion). A greater surface area and an increased number of amino acids participate in the interaction between β3 and αIIb compared to αv both for the domain containing the β3P163S mutation (A.1 and B.1 windows) and in the entire complex between the headpieces. Significantly, as well as β3P163, amino acids engaged in H-bonds within αIIbβ3 and αvβ3 are highly conserved through species (Figure S2) although to a lesser extent within different α-subunits of the integrin family in man. Interestingly, this initial analysis highlighted only a single H-bond between β3P163 and H113 in αv (see Box B1).

### Molecular Dynamics Analysis

**Figure 4:**
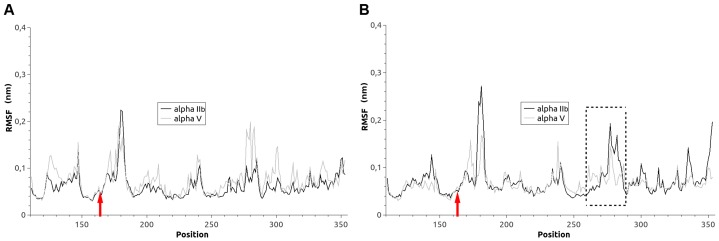
Effect of the β3P163 substitution on the backbone flexibility of β3 within the integrin complex. RMSF values are calculated for each residue within WT β3 (A) and P163S β3 (B) in complex with either αIIb (heavy line) or αv (faint line). Red arrows indicate the position of the mutation. The largest changes are seen approximately 100 amino acids forward from the mutation (dotted box).

We then used molecular dynamics simulations to examine the effects of the P163S substitution on αIIbβ3 and αvβ3 structure. We first plotted the RMSD (root mean square deviation) for each C-α position of wild type (WT) or P163S substituted β3 in complex with αIIb or αv (Figure 4). Both integrin complexes show equivalent movements of the β3 backbone. The introduction of S163 induced only small changes in fluctuations at the site of the mutation (red arrows) for β3 in complex with αIIb or with αv. However, more substantial changes occur approximately 100 amino acids onward with a dramatic increase in movements when mutated β3 is in complex with αIIb and, in contrast, a decrease and stabilization of the backbone structure when mutated β3 is associated with αv (dotted box).

**Figure 5:**
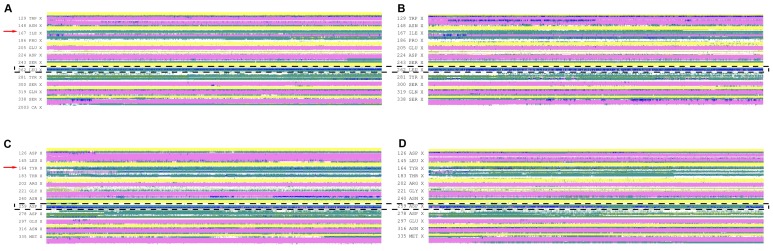
Timeline plots of the β3 secondary structure. Illustrated are the WT form (A, C) and the P163S mutated form (B, D) either associated with αIIb (A, B) or αv (C, D) subunits. Shown are molecular dynamics time and primary sequence: time (60 ns) is on the horizontal axis and primary sequence is on the vertical axis. The following color code is used for the secondary structure: dark green = turn, yellow = β-sheet, pink = α-helix, blue = 3–10 helix, red = pi-helix, white = random. Position 163 is indicated by red arrows and the region concerned by the major changes is framed with a dotted box.

The influence of the P163S mutation on the secondary structures of β3 in complex with αIIb or αv was then examined in timeline plots (Figure 5). From left to right of these plots it is possible to follow the influence of the mutation on the secondary structure from the beginning (on the left) to the end of the dynamics run (after 50 ns of full dynamics). The main secondary structures (alpha helices in magenta and beta-strands in yellow) are largely unaffected by the mutation. Again, while the β3 secondary structure at the site of the substitution appears unaffected (red arrows), changes occur around 100 amino acids onwards (blue dotted box). Significantly, while a 3–10 amino acid α-helix (in blue) beginning at position 259 and framed by two β-turns (in green) can be clearly distinguished in WT αIIbβ3, the presence of S163 results in a loss of the last β-turn. This latter structure is also lost when β3 is associated with αv. Other differences are the loss of a small α-helix around position 229 for the α-subunit in αvβ3 and the appearance of a small α-helix around position 170 for the β-subunit in αvβ3. A 3–10 amino acid α-helix visible between position W129 and N148 was transient in nature when mutated β3 was in complex with αIIb and was lost after 10 to 15 ns of molecular dynamics, its significance is unknown.

In the WT integrins, the nature of the α-subunit clearly has a significant influence on the number of hydrogen bonds engaged by β3 (Table I) and confirms the static analysis. As measured in the last 6 ns of the molecular dynamics runs, in comparison to αIIbβ3, αvβ3 shows a small global increase in H-bonds (+2.5%) and at the same time a marked decrease in the number of inter-subunit H-bonds (−23.3%). This suggests either a decreased stability of WT αvβ3 compared to αIIbβ3 or a weaker binding between the subunits in αvβ3. With β3S163, the consequences are different. For αIIb there is a moderate increase (+11%) in inter-subunit H-bonds and a small reduction in the global H-bonds network (- 3.4%). However, for αvβ3 the mutation induces a dramatic increase (+41.5%) in inter-subunit H-bonds but a negligible decrease (- 0.6%) in the H-bond global network (Table I). Overall, compared to the WT proteins, the P163S mutation induces a straightening of the inter-subunit domain that is slight with αIIbβ3 but extensive with αvβ3. In the same way, global RMSD analyses of the complexes throughout 60 ns of molecular dynamics revealed β3 rearrangements that were modest with αIIb (black and green traces) and profound with αv (red and blue traces) (Figure S3).

**Table 1:** Hydrogen bond changes in the different models.

	αIIbβ3 WT	αvβ3 WT	αIIbβ3 P163S	αvβ3 P163S
**Average number**				
H-bonds (inter)	5.28	4.05	5.86	5.73
H-bonds (intra)	150.5	155.6	144.6	152.9
Global	155.78	159.65	150.46	158.63
**% change vs WT αIIbβ3**				
H-bonds inter		−23.3%	11.0%	
H-bonds intra		3.4%	−3.9%	
Global		2.5%	−3.4%	
**% change vs WT αvβ3**				
H-bonds inter				41.5%
H-bonds intra				−1.7%
Global				−0.6%

Average number of (inter-, intra- or global) H-bonds found during the last 6 ns of the molecular dynamics simulations.

In terms of individual bonds, in αIIbβ3 the newly introduced S163 on β3 exchanges strong H-bonds with E168 on the α-subunit. This contrasts with the WT complex where E168 exchanges H-bonds essentially with A263 on β3 and W110, P126 and F171 on αIIb all of which are lost in the presence of the mutation. Moreover, the new S163 now also exchanges H-bonds with R216 and L262 on β3. P163 does not appear in the WT H-bond list and neither do R216 and L262. P163 is also not in the H-bond list for WT αvβ3 where the introduced S163 now forms weak H-bonds with L262 and N156 on αv. The main H-bonds in WT αvβ3 are between D259 (β3) and Y275 (αv) S291 (β3) and E311 (αv) and between T296 (β3) and L309 (αv). In the presence of S163, the interaction between S291 (β3) and E311 (αv) is much stronger while that between Y275 (αv) and D259 (β3) is weaker. Several new interactions appear: S300 (β3) with D306 (αv), D259 (β3) with Y221 (αv) and Y166 (β3) with Y178 or E121 (αv).

Major changes also occur for β3R261 that exchanges H-bonds with a number of amino acids on αIIb in the WT integrin: Y237, A95, F21, F419, W110, G170, F171 and Y288 while only forming H-bonds withY237 and F171 in the mutated form (Figure S4). For αv, the number of H-bonds involving β3R261 shows little change although they involve different partners: Y224, Y406, F278, Y406 and Y224 in the WT form; and Y406, F178, F159, Y224 and A96 in the presence of β3S163.

### Summary of the Effects of the P163S Substitution

**Figure 6:**
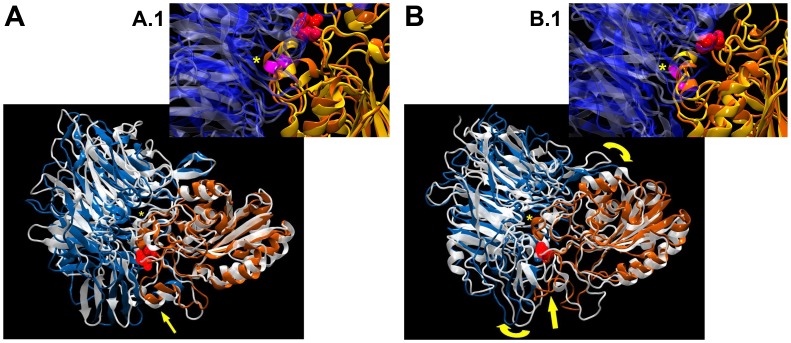
Summary of the major changes seen in the molecular dynamics runs. Position 163 on β3 is shown as red spheres. A/Superimposition of the two forms of αIIbβ3: αIIb associated with WT β3 is in silver glass ribbon while blue ribbon highlights αIIb in complex with the β3P163S mutant; orange ribbon denotes β3. Note that the mutation induces only a slight displacement of the small α-helix close to position 163 (yellow arrow below) and a larger change for the small α-helix beginning at position 259 but outside of the interface (yellow*). B/Superimposition of the two forms of αvβ3: αv associated with WT β3 is in silver glass ribbon while blue ribbon shows αIIb associated with the β3P163S mutant; orange ribbon denotes β3. Note the unfolding of the small α-helix close to position 163 (yellow arrow below), the large displacement towards the interface of the α-helix beginning at position 259 on β3 (yellow*) and the new fold appearing in the P163S mutant (yellow arrow) that is projected towards the interface resulting in a clockwise rotation of the αv sub-unit (yellow curved arrows). Windows correspond to a zoom of the regions marked by the asterisk.

The major structural changes are highlighted when the WT and mutant forms of both αIIbβ3 and αvβ3 are superimposed (Figure 6). For αvβ3 note the unfolding of the small α-helix close to position 163 (between 169 and 174, lower yellow arrow, Figure 6B); a displacement towards the interface of the α-helix beginning at position 259 on β3 (yellow*) and the new fold appearing in the P163S mutant (between 166 and 174, yellow arrow) that projects toward the interface resulting in a clockwise rotation of the αv subunit (yellow curved arrows). Comparison of the two forms of αIIbβ3 (Figure 6A) shows that the mutation only slightly displaces the small alpha-helix close to position 163 (lower yellow arrow) but results in the small alpha-helix beginning at position 259 (yellow*) moving away from the interface (approximately −3.5 Å). This 3_10_-α-helix contains β3R261 that is now localized 3.5 Å outside of the αIIb headpiece when compared to the WT conformation. In contrast, β3R261 sinks deep into the β-propeller of the αv headpiece (approximately 4.4 Å in comparison to the WT conformation).

## Discussion

The crystal structure of the extracellular segment of integrin αvβ3 provided the first clear insights into the extracellular head domain structure and how conformation changes with the activation state of the integrin [14]–[19]. Close contacts between the two subunits primarily involved the αv β-propeller and the β3 β-I (βA) domains. β-I also contains functionally important MIDAS and ADMIDAS sequences with 3 metal ion-binding domains. A key residue is β3R261 that lies at the core of the β-I domain-β-propeller interface and is surrounded by two concentric rings of predominantly aromatic α-subunit β-propeller residues. Side-chains of F21, F159, Y224, F278 and Y406 from the lower ring were said to interact with R261 directly. Residues Y18, W93, Y221, Y273 and S403 in the upper ring contact side-chains in the lower ring and provide a hydrophobic interface for residues flanking β3R261 in the so-called 3_10_-α-helix [14]. Additional contacts were also shown between more distant parts of the head domains of both subunits. It was noted even at this early time that β3P163, the amino acid mutated in our patient, lies in a loop adjacent to the 3_10_-helix of αv.

Homology models were first used to extrapolate results for αvβ3 to αIIbβ3 and predict contact interactions between αIIb and β3. These became redundant when a refined crystal structure of the complete αIIbβ3 ectodomain obtained in the presence of Ca^2+^ and Mg^2+^ permitted direct analyses [16]–[19]. Water molecules that favor hydrogen bonding and metal coordination were located in the αIIbβ3 but not the αvβ3 structure. Particularly highlighted were three non-conserved loop region structures (residues 71–85, 114–125 and 148–164) of human αIIb while K118 was said to form a salt bridge with E171 in the specificity-determining loop (SDL) of β3 (residues 159–188) that contains P163. The structural importance of these residues is highlighted by the large number of missense mutations in the β-propeller region of αIIb detected in patients with classic type I GT [2], [3], [27]. Crystallography also predicted that αIIb residues L116, K124 and R153 were close to one or more residues of the β3 SDL region. β3 residues I167, S168 and P169 were said to have a side chain or backbone within 5 Å of αIIb residues. Further proof for residues in close contact came from a cysteine substitution model that provoked the formation of disulfide-linked dimers when the mutated αIIb and β3 subunits were transfected into HEK 293 cells [16].

It is in this context that we now report a GT patient with a β3 P163S substitution with little or no expression of αIIbβ3 at the platelet surface but with residual β3 and a usual presence of αvβ3 in her platelets. Transfection of WT and mutated integrins in CHO cells recapitulated the loss of cell surface expression of αIIbβ3 in cells co-transfected with WT αIIb and β3S163, and confirmed the capacity of the mutated β3 to bind endogenous hamster αv and form a heterodimer that was transferred to the cell surface. Notwithstanding, differences in surface expression of chimeric αvβ3 were observed (Figure 2) depending on the use of a monoclonal antibody to human β3, which detected similar levels of αvβ3 in cells transfected with the β3S163 variant compared to those transfected with WT β3, or a monoclonal antibody to human αvβ3, which showed a reduced expression between the WT αvβ3 and the αvβ3S163. The latter most likely reflects differences in the ability of hamster αv to bind to WT β3 and β3S163 or to conformational changes within the epitope recognized by the LM609 antibody. For platelets of the patient, αvβ3 had a mostly vesicular localization as previously described by us for normal platelets and for those of another type I GT patient with a homozygous *ITGA2B* E324K mutation and residual β3 [8]. The reason for this localization is unknown but is consistent with a trafficking role for αvβ3; roles for αvβ3 in transport of vitronectin and in the sensing of bacterial lipopeptides have been previously described [28], [29]. As this was not a major thrust of our paper the localization of αvβ3 was not studied further. Significantly an adjacent β3S162L mutation was previously reported in a GT patient with much decreased amounts of platelet αIIbβ3; S162 lies close to blade 2 of the propeller and its replacement by L162 results in unfavorable contacts at the αIIb and β3 interface underlining the structural importance of this particular β-I domain; αvβ3 was not studied by the authors [27], [30].

Computer modeling and a molecular dynamics analysis confirmed that the P163S mutation affected the β3 interface with both αv and αIIb and showed the advantages of the dynamic approach in evaluating the structural effects of amino acid substitutions. The previously detected salt bridge between αIIbK118 and β3E171 (16–18) was maintained at least partly in the WT integrin during the molecular dynamics run; but for the P163S mutant and due to the relative movements of the two subunits it was replaced by a salt bridge between β3E171 and αIIbR122. Globally, αIIbβ3S163 showed a moderate 11% increase in intra-subunit H-bonds and a very weak decrease in the global H-bond network but αvβ3S163 showed a dramatic 41% increase in intra-subunit H-bonds without modifying the H-bond global network. Compared to the WT proteins, the P163S mutation induces a straightening of the inter-subunit interactions that is slight with αIIbβ3 but extensive for αvβ3. These structural rearrangements result in positioning of β3R261 outside the β-propeller in αIIbβ3 but deep inside for αvβ3. All in all, mutated αvβ3 appears to have an increased stability perhaps confirmed by the intensity of the residual β3 band observed in the patient’s platelets by Western blotting.

Molecular dynamics simulations and modeling of αIIbβ3 have recently been reported for a homozygous αIIb N2D mutation present in 4 siblings of an Israeli Arab family that affects blade 1 of the β-propeller [31]. There was no surface expression of αIIbβ3 in platelets or after transfection of the mutated integrin in BHK cells; the mutated pro-αIIbβ3 complex was formed but trafficking was impaired. N2 is surface exposed on the β-propeller and is highly conserved. Here, a H-bond between N2 and L366 of a calcium-binding domain in blade 6 of αIIb was disrupted, thereby impairing calcium binding essential for intracellular trafficking of pro-αIIbβ3. When the equivalent mutation was introduced into αvβ3 it had a less deleterious effect in transfected BHK cells confirming a lower sensitivity of αvβ3 to calcium chelation. Molecular dynamic simulations of the wild-type and mutant proteins indicated that aa364–370 fluctuated more in the mutant αIIb with a shifting out of blade 6 [31].

Other mutations that differentially affect αIIbβ3 and αvβ3 include a L196P mutation adjacent to the β3 MIDAS (amino acids 118–131) in two French GT patients that allowed residual (10 to 15%) expression of non-functional αIIbβ3 [10], [11]. Transfection of β3P196 with wild-type αIIb in CHO cells confirmed interference with αIIbβ3 maturation yet αvβ3 was normally expressed [10]; a result similar to that now reported by us for β3P163S. A β3 L262P mutation gave residual αIIbβ3 able to bind fibrin and with platelets able to retract clots; yet the platelets did not bind Fg when stimulated [32]. Leu262 occurs within an intrachain disulfide loop (between C232 and C273) important for subunit assembly and is joined to β3R261 in the 3_10_-α-helix. When transiently transfected with wild-type αIIb in COS-7 cells, αIIbβ3P262 allowed normal heterodimer formation but export from the endoplasmic reticulum was delayed and those complexes that reached the surface were unstable. β3P262 transfected in human embryonic kidney 293 cells formed a complex with αv and retracted fibrin clots although the cells did not interact with immobilized Fg.

As we have reviewed elsewhere, other mutations within β3 mimic β-I domain P163S by differently affecting αIIbβ3 and αvβ3 expression [33]. These include breakage of some of the 56 disulfides in the EGF domains of β3 [13], [34]–[36]. For example, disrupting C473–C503 caused reduced surface expression of αvβ3 relative to αIIbβ3 whereas disruption of C437–C457 by C457S resulted in a significant reduction of αIIbβ3 compared to αvβ3 [13]. Molecular dynamics analysis was performed using a mutated β3 fragment composed of the four EGF domains and β-tail domain derived from αIIbβ3 and αvβ3 crystal structures [13]. The mutated αIIbβ3 structure was changed considerably from the native one and was stable in a new activated conformation whereas the final αvβ3 structure resembled the starting conformation.

Another mutation in β3 exerting a more deleterious effect on αIIbβ3 than αvβ3 expression is H280P (variant Osaka-5). H280P was found in three unrelated Japanese patients (one homozygous and two heterozygous) with residual αIIbβ3 expression [9], [37]. Platelets expressed about half the normal amounts of αvβ3 whereas αIIbβ3 levels were reduced to about 6%.

Taken in this context, our studies on β3 P163S provide new evidence as to how missense mutations within the extracellular domain of β3 can differentially influence αIIbβ3 and αvβ3 expression. We show how β3S163 affects the three-dimensional structure of the integrins differently and that αvβ3 can even become more stable. This has important implications for considering genotype/phenotype relationships in Glanzmann thrombasthenia. Up-to-now, no clear differences in phenotype have been reported between patients with *ITGA2B* or *ITGB3* mutations [2], [3], [38]. However, the structural consequences of *ITGB3* missense mutations are clearly variable and therefore it is necessary to establish for each patient how αIIbβ3 and αvβ3 are affected. In this respect, the human disease differs from mouse models where the *Itgb3* gene is specifically deleted [2].

## Supporting Information

Figure S1 **Flow cytometry measuring the binding of selected monoclonal antibodies to platelets of the patient.** This study was performed according to our standard procedures using a Becton Dickenson FACScan [39], [40]. Note the minimal binding of AP2 (anti αIIbβ3) and Tab (anti- αIIb); a slightly higher binding of AP3 (anti-β3) and a normal binding of BX1 (anti-GPIbα) to the platelets of the patient.
(TIF)

Figure S2 **Conservation of β3 Pro163.** Residue P163 (*) of β3 is highly conserved within mammals and vertebrates (A) and within different integrin β-subunits in man (B). Also shown is the highly conserved nature of αIIb amino acids (C) and of αv amino acids (D) forming H-bonds with β3P163. In dotted boxes are amino acids participating in H-bonds within αIIbβ3 but not within αvβ3.
(TIF)

Figure S3 **Molecular dynamics analysis.** Plots of RMSD *vs*. time of the global integrin complex of the αv and β3 subunit headpieces during a complete (60 ns) molecular dynamics run. Shown are the results for wild-type αIIbβ3 and αvβ3 and for αIIbβ3S163 and αvβ3S163.
(TIF)

Figure S4 **3D-modelisation of amino acids interacting with β3.** Amino acids are represented as sticks; β3R261 is coloured in magenta while amino acids from αIIb or αvβ3 are coloured in dark green for the wild type integrin and in pink and light green for the mutated form. The initial position for β3R261is superimposed as a transparent image.
(TIF)

Case History S1 (DOCX)
