# Supplementary figures and images for: Molecular Dynamics Analysis of a Novel β3 Pro189Ser Mutation in a Patient with Glanzmann Thrombasthenia Differentially Affecting αIIbβ3 and αvβ3 Expression
Source: PLoS One. 2013 Nov 13;8(11):e78683. doi: 10.1371/journal.pone.0078683 (PMC3827234; doi:10.1371/journal.pone.0078683)

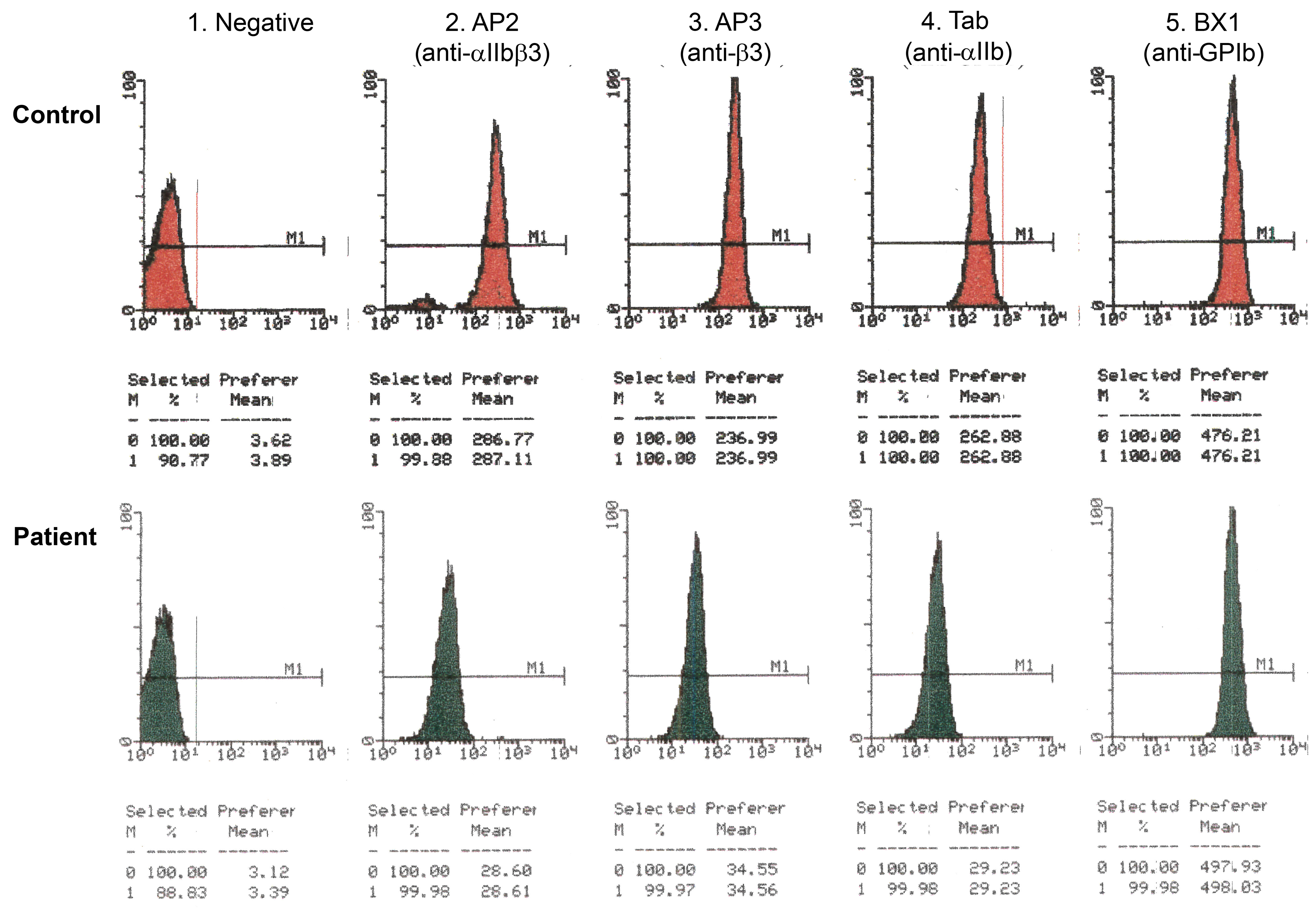

Supplement: Figure S1 — Flow cytometry measuring the binding of selected monoclonal antibodies to platelets of the patient. This study was performed according to our standard procedures using a Becton Dickenson FACScan [39], [40]. Note the minimal binding of AP2 (anti αIIbβ3) and Tab (anti- αIIb); a slightly higher binding of AP3 (anti-β3) and a normal binding of BX1 (anti-GPIbα) to the platelets of the patient. (TIF) [file pone.0078683.s001.tif]

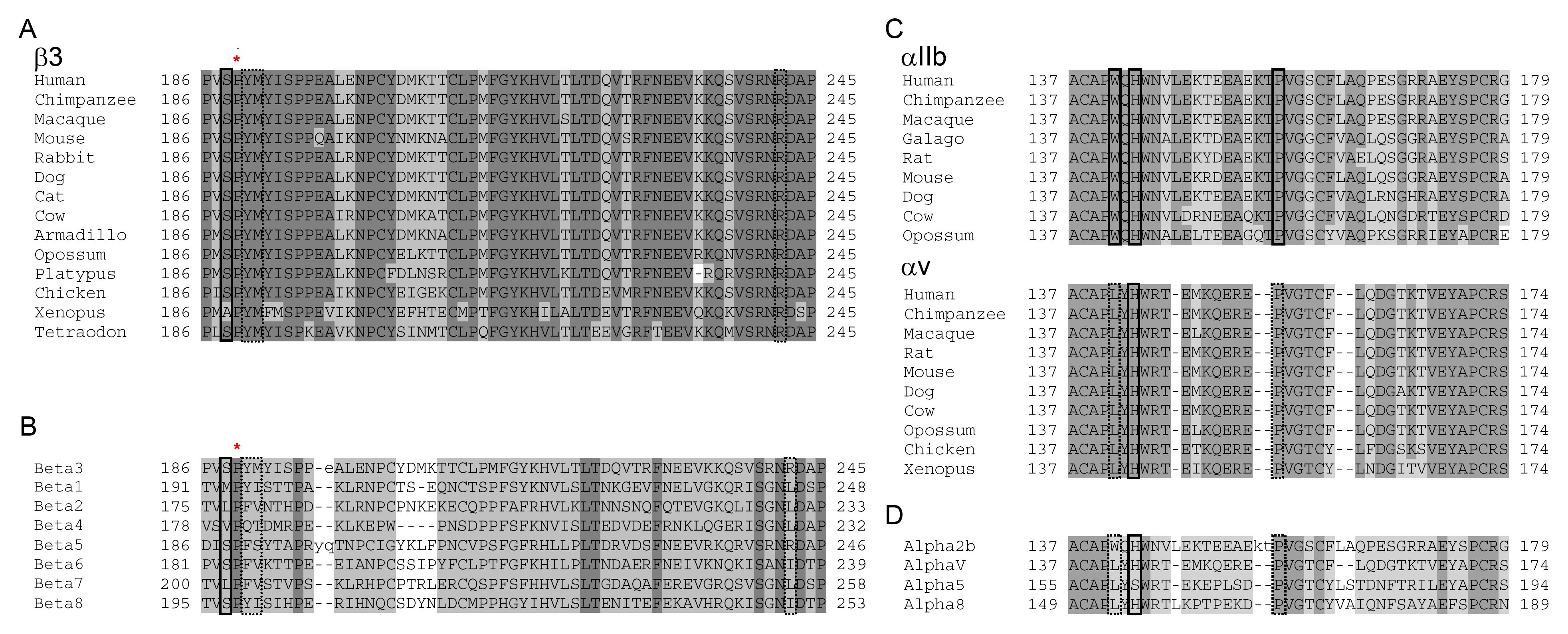

Supplement: Figure S2 — Conservation of β3 Pro163. Residue P163 (*) of β3 is highly conserved within mammals and vertebrates (A) and within different integrin β-subunits in man (B). Also shown is the highly conserved nature of αIIb amino acids (C) and of αv amino acids (D) forming H-bonds with β3P163. In dotted boxes are amino acids participating in H-bonds within αIIbβ3 but not within αvβ3. (TIF) [file pone.0078683.s002.tif]

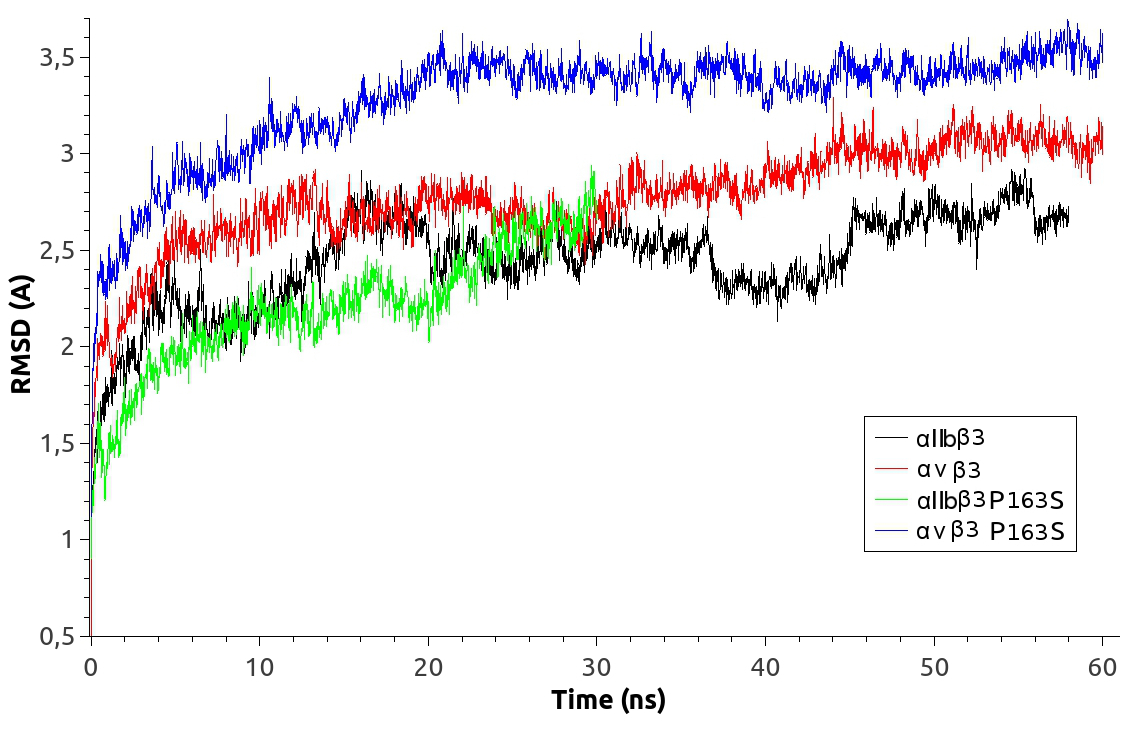

Supplement: Figure S3 — Molecular dynamics analysis. Plots of RMSD vs. time of the global integrin complex of the αv and β3 subunit headpieces during a complete (60 ns) molecular dynamics run. Shown are the results for wild-type αIIbβ3 and αvβ3 and for αIIbβ3S163 and αvβ3S163. (TIF) [file pone.0078683.s003.tif]

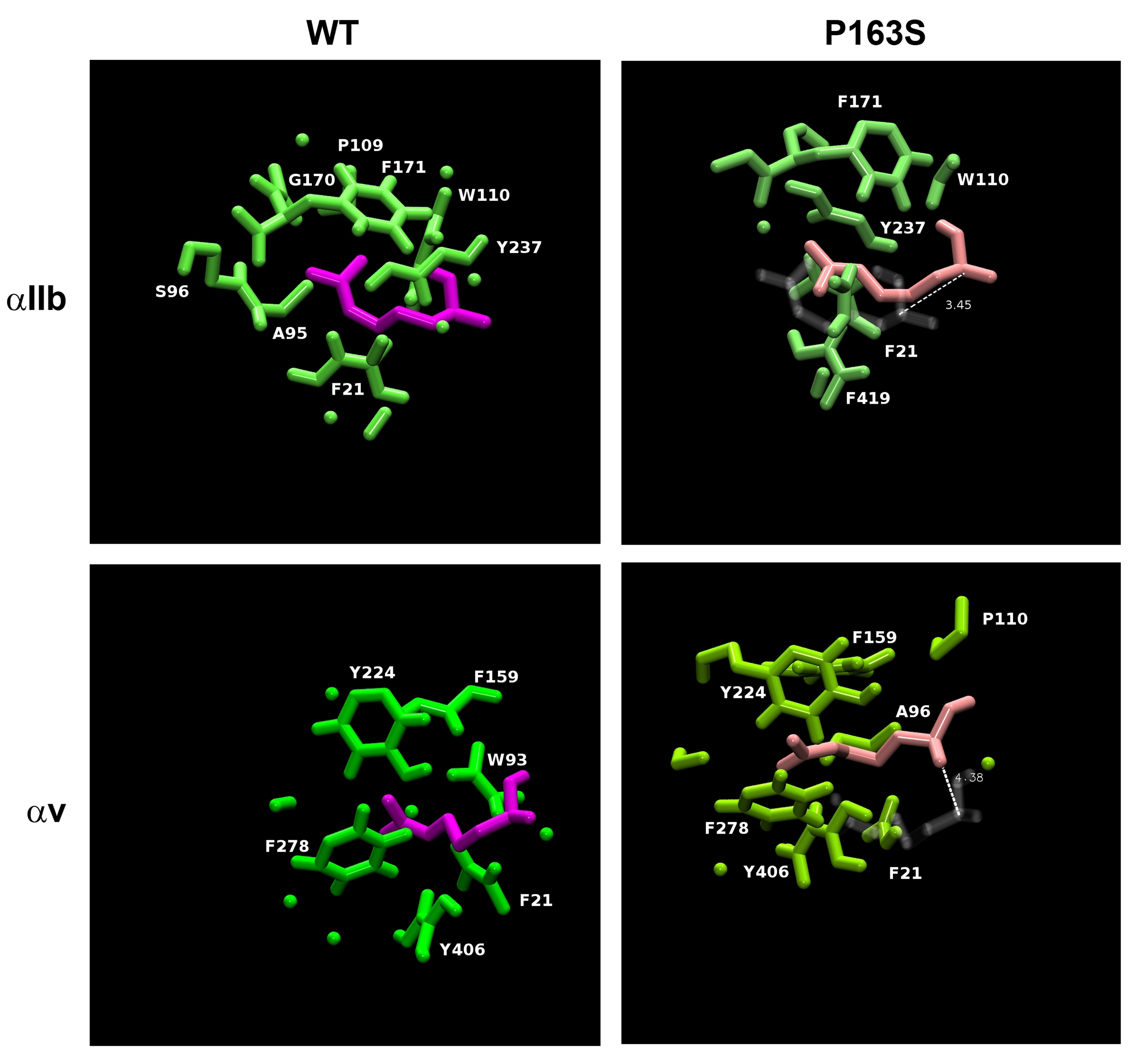

Supplement: Figure S4 — 3D-modelisation of amino acids interacting with β3. Amino acids are represented as sticks; β3R261 is coloured in magenta while amino acids from αIIb or αvβ3 are coloured in dark green for the wild type integrin and in pink and light green for the mutated form. The initial position for β3R261is superimposed as a transparent image. (TIF) [file pone.0078683.s004.tif]
