# Supplementary material for: Molecular Dynamics Analysis of a Novel β3 Pro189Ser Mutation in a Patient with Glanzmann Thrombasthenia Differentially Affecting αIIbβ3 and αvβ3 Expression
Source: PLoS One. 2013 Nov 13;8(11):e78683. doi: 10.1371/journal.pone.0078683 (PMC3827234; doi:10.1371/journal.pone.0078683)
Supplement: Case History S1 — (DOCX) [file pone.0078683.s005.docx]

The patient is a 49 year-old French woman from a consanguineous family, she was diagnosed with GT when 5 years old. A brother died from haemorrhage when 4 years old but an adult sister has never experienced untoward bleeding. As a girl, the patient suffered from easy bruising and repeated epistaxis that often required platelet transfusions. Severe menstrual bleeding at menarche was controlled by birth control pills. During adolescence her bleeding was principally gingival and dental requiring hospital treatments. Mucocutaneous bleeding has become less frequent since becoming adult although hematuria was noted in 1991. Her platelet count is relatively low (e.g. 136 x 10^8^/ml and 162 x 10^8^/ml; control range 150 – 400 x 10^8^/ml) but platelet volumes have always been normal. Platelet function testing confirmed an absence of aggregation to ADP, collagen, arachidonic acid and thrombin but the response to ristocetin was normal, findings typical of GT [1]. There was no clot retraction. The PFA-100 occlusion time when tested with the epinephrine/collagen cartridge was > 300s. Anti-platelet antibodies specific for αIIbβ3 were detected in 1994 and these were shown to block the aggregation of platelets from a control donor [38]. However, the antibodies were no longer present in 2006. Standard flow cytometry analysis showed no binding of P2, and AP-2, two MoAbs recognizing αIIbβ3 and a very weak binding of MoAbs to β3 to platelets of the propositus (Figure 1 S1). When the platelets were activated with thrombin, P-selectin was normally expressed at the platelet surface.
